# Software Application Profile: TIDAL—Tool to Implement Developmental Analysis of Longitudinal data

**DOI:** 10.1093/ije/dyaf095

**Published:** 2025-07-06

**Authors:** Alex S F Kwong, Amelia Edmondson-Stait, Eileen Xu, Ellen J Thompson, Richard M A Parker, Ahmed Elhakeem, Liana Romaniuk, Rebecca M Pearson, Kate Tilling, Thalia C Eley, Andrew M McIntosh, Heather C Whalley

**Affiliations:** Division of Psychiatry, Centre for Clinical Brain Sciences, University of Edinburgh, Edinburgh, United Kingdom; Population Health Sciences, Bristol Medical School, University of Bristol, Bristol, United Kingdom; MRC Integrative Epidemiology Unit, University of Bristol, Bristol, United Kingdom; Division of Psychiatry, Centre for Clinical Brain Sciences, University of Edinburgh, Edinburgh, United Kingdom; Division of Psychiatry, Centre for Clinical Brain Sciences, University of Edinburgh, Edinburgh, United Kingdom; Faculty of Science, Engineering and Medicine, School of Psychology, University of Sussex, Brighton, United Kingdom; Social, Genetic and Developmental Psychiatry, King’s College London, London, United Kingdom; Population Health Sciences, Bristol Medical School, University of Bristol, Bristol, United Kingdom; MRC Integrative Epidemiology Unit, University of Bristol, Bristol, United Kingdom; Population Health Sciences, Bristol Medical School, University of Bristol, Bristol, United Kingdom; Division of Psychiatry, Centre for Clinical Brain Sciences, University of Edinburgh, Edinburgh, United Kingdom; Department of Psychology, Manchester Metropolitan University, Manchester, United Kingdom; Population Health Sciences, Bristol Medical School, University of Bristol, Bristol, United Kingdom; MRC Integrative Epidemiology Unit, University of Bristol, Bristol, United Kingdom; Social, Genetic and Developmental Psychiatry, King’s College London, London, United Kingdom; Division of Psychiatry, Centre for Clinical Brain Sciences, University of Edinburgh, Edinburgh, United Kingdom; Division of Psychiatry, Centre for Clinical Brain Sciences, University of Edinburgh, Edinburgh, United Kingdom

**Keywords:** longitudinal, trajectories, multilevel growth-curve modelling, Millennium Cohort Study, ALSPAC

Key Features

- TIDAL is a free and easily accessible research tool for analysing, visualizing and interpreting growth curve model data.
- TIDAL can plot complex, non-linear trajectories and aid in interpretation of those trajectories throgh features such as trajectory visualization, calculating scores at different ages and the area under the curve.
- TIDAL can examine interactions between trajectories and continuous or categorical covariates to obtain group specific trajectories.
- TIDAL is available in three formats: an R package, a Docker Image and an online RShiny application.

## Introduction

Growth-curve modelling uses repeatedly measured data from the same individuals to estimate trajectories that describe how variables change over time and identify key periods of change and why they occur [1, 2]. This method is used in life-course epidemiology and related disciplines including psychology, social science, and public health [3, 4]. Of particular interest is examining trajectories according to key potential effect modifiers. Some examples include estimating trajectories of height and how they vary by sex [5], body mass index (BMI) trajectories stratified by levels of social adversity [6], cross-cohort differences in bone mineral content (BMC) trajectories [7], and depression trajectories stratified by genetic risk [8].

Multilevel growth-curve modelling (MLM, also known as hierarchical modelling or mixed-effects modelling) [9, 10] is one method for estimating trajectories and reflects the fact that longitudinal data have a hierarchical or multilevel structure, with repeated observations (i.e. of height) nested within individuals [2]. This nesting means that the observations are not independent and analyses that ignore this clustering can result in inappropriate standard errors and potentially erroneous results. MLM is a popular and flexible method that can be applied to individuals throughout the life course [4]. Leveraging a recent influx of longitudinal data and applying growth-curve methods could enhance our understanding of longitudinal traits and behaviours across development, potentially leading to better interventions and preventions.

However, there are barriers in place that prohibit the successful use and implementation of trajectory modelling. Whilst most statistical software packages accommodate growth-curve analysis including Stata, R, SPSS, Python, and SAS, they are not all accessible to users and many require time, costs, and expertise to successfully implement and interpret. For example, users without statistical backgrounds may find it difficult to use some statistical software or not have the capacity to learn new methods (time or costs to train), despite in-depth knowledge of the trait that they are interested in. Additionally, whilst some applications and resources exist that facilitate trajectory modelling [11, 12], they all require statistical and software expertise to successfully implement and are not necessarily accessible and interpretable for naïve users. Finally, and perhaps more importantly, even experienced users may struggle to interpret more complex trajectories, which is especially the case with non-linear trajectories that often result in estimates that are not easily interpretable [13].

To address this, we have developed TIDAL (Tool to Implement Developmental Analysis of Longitudinal data)—a free and easily accessible research tool for data wrangling, analysing, visualizing, and interpreting growth-curve model data aimed at a variety of users, including researchers, clinicians, public health officials, government analysts, and educators.

## Implementation

The TIDAL application can be found at https://tidal-modelling.github.io. The tool is currently available in three formats: an R package, a Docker Image, and an online RShiny application. The R package and Docker Image can be downloaded and installed locally and used offline, allowing users to analyse potentially sensitive data locally without uploading data to online servers.

The TIDAL website contains an overview of the tool, five example datasets, detailed instructions, and a guided tutorial on how to install, run, and interpret TIDAL (in video and PDF formats), a frequently-asked-questions section, and some guidance for further information on statistics and growth-curve modelling.

**Figure 1.**
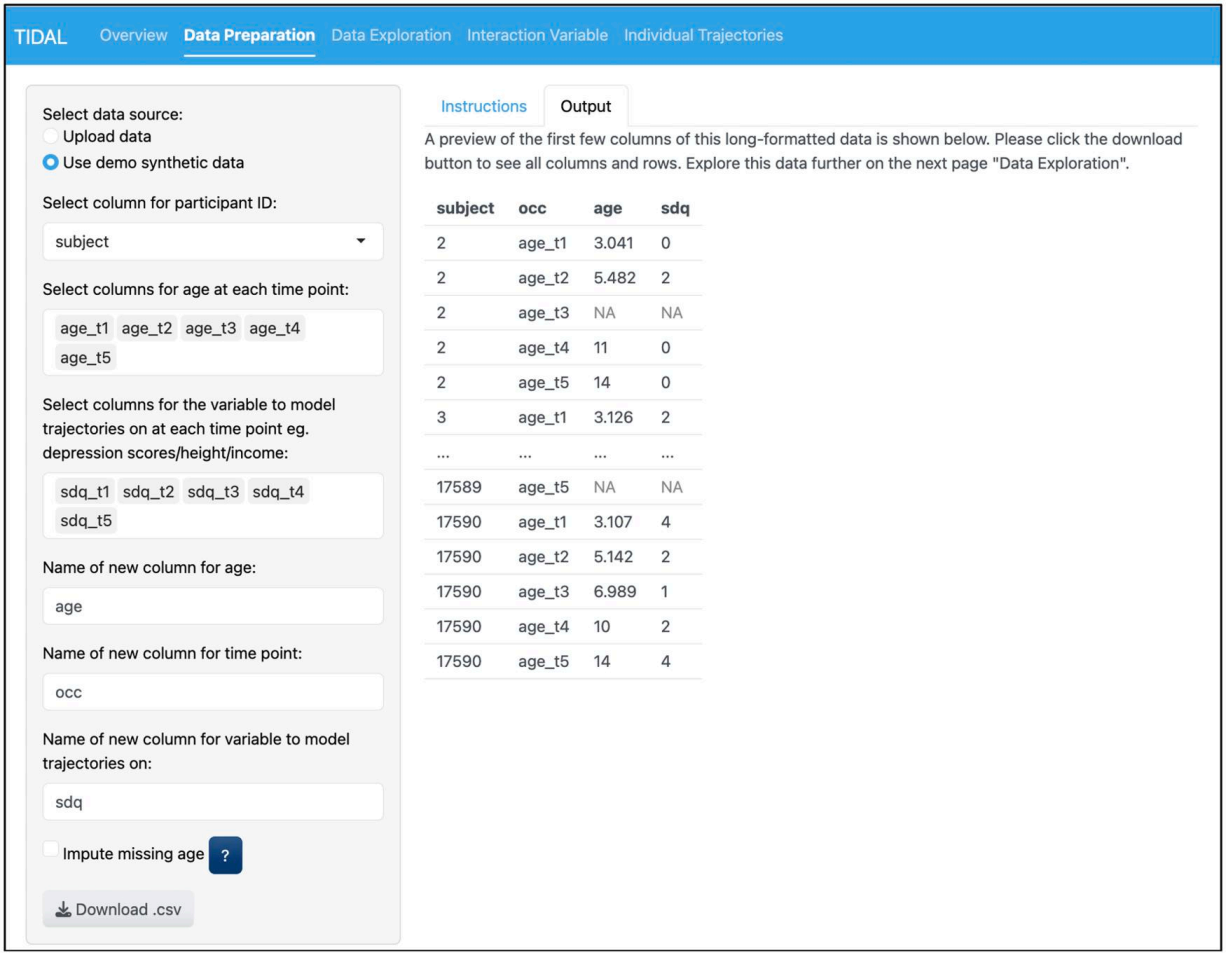
The data-preparation stage for TIDAL. This figure shows the data-preparation stage by using the built-in example data in TIDAL. The first three dropdown options include the variables subject, age_t1 to age_t5, and sdq_t1 to sdq_t5, which are selected from the original wide dataset. The bottom three dropdown options can all be renamed. Here, the defaults of time_point and score have been renamed as occ and sdq, respectively. On the right, a table under the options tab shows the new data structure in long format.

**Figure 2.**
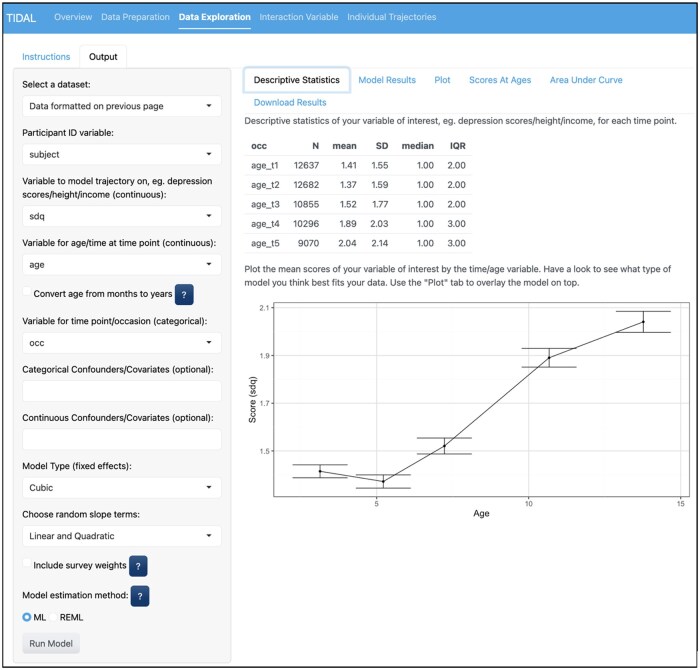
The data-exploration and analysis stage of TIDAL. This figure shows the data-exploration stage by using the formatted data from the previous stage (highlighted by the first drop box). The variables sdq, age, and occ are all selected as outcome and time variables, respectively, carried forward from the previous stage. The right side shows the descriptive statistics and a plot of the mean scores by age/time to guide the choice of trajectory modelling. In this example, the data appear to represent a non-linear trajectory and a cubic model is chosen from the Model Type dropdown box with random linear and quadratic slope terms.

We have developed TIDAL to guide users through the main steps involved in growth-curve modelling analysis. These include:

- Data import and preparation: the first stage of TIDAL is to import the data. TIDAL accepts comma separated .csv or tab delimited .txt or .tsv files to the limit of 400 MB. These data would typically be continuous variables such as BMI assessments across four occasions, with corresponding age and covariate information, or mental health scores across five waves split by treatment arms. Missing values should be set to ‘NA’ or empty cells (‘’). A synthetic dataset is embedded within the tool for exploratory use and analysis. TIDAL will convert data from wide (one row per individual) to long (one row for each measurement occasion within each individual) format to accommodate multilevel growth-curve modelling. This step can be bypassed if the user already has a long-format dataset.
     If the user imports wide-format data, then they are first required to select the identifier/subject, repeated ages/times/occasions, and repeated outcomes variables from the dropdown boxes provided (see Fig. 1 for an example). Variables are then created for a new long-format dataset to be analysed and users can rename these from the default to more relevant names. A snapshot of these new long-format data is presented for users to ensure that their data have been formatted correctly, with error messages appearing if an unequal number of occasions and outcome variables have been entered (i.e. four occasion rows but only three outcome rows). Users can download the newly formatted long dataset for analysis outside of TIDAL if desired.
- Data exploration and analysis: the next stage of TIDAL uses the newly formatted long dataset from the previous stage or a new user uploaded a long dataset to begin exploring the data. Users are required to select the relevant names from the dropdown boxes corresponding to the identifier/subject, outcome, and age/time/occasion variables, respectively (see Fig. 2).

    Users can then select the type of growth-curve model from a choice of linear and non-linear functions to describe the association of the outcome with time/age and can specify fixed (population) and random (individual) effects. The lme4 package in R is used to conduct the growth-curve modelling [14]. Missing outcome data are handled by using Maximum Likelihood (ML) or Restricted Maximum Likelihood (REML), which, in short, use all observed data to estimate the model parameters without the need for imputation of missing data. Individuals with missing covariate data are excluded through listwise deletion. Further information regarding the statistical properties of these models is given in the Supplementary Materials. Currently, TIDAL supports trajectory modelling by using fixed linear, quadratic, cubic, and quartic polynomial terms, and random intercept, linear, and quadratic terms (allowing random slopes), as per previous research [1, 7, 15]. To guide the choice of modelling, a line plot of the mean scores at each occasion is presented, alongside descriptive information (*N*, mean, standard deviation, median, and interquartile range of the outcome variable at each occasion). Users need to select a model to initiate the descriptive information and plot, so we recommend running a linear model to begin exploring the data. Users can include continuous or categorical covariates, survey weights into their models, and choose which estimator to use: ML (the default) or REML. Continuous covariates are averaged across the values of that covariate within the sample, whereas categorical covariates are set to their lowest level when included into the model. Information on these is given on the TIDAL application and in the Supplementary Materials.

   The time taken to run a model will depend on several factors. Models with many participants (>10 000), many repeated assessments (>5), complex missing data patterns, or an increasing number of fixed and/or random effects will result in greater model complexity and longer waiting times. However, most models should complete in <30 seconds based upon our testing across datasets and traits. Should the model not complete or fail to converge, error messages highlighting the underlying issue will appear.

   Once the model completes, the lme4 syntax used to run the model in R will be presented. Users could adapt this syntax outside of TIDAL to create more complex models. The model estimates are presented giving the effect sizes, 95% confidence intervals (CIs), and *P*-values, alongside a value for model fit (deviance). A table of estimated variances and covariances of random effects is also presented. Importantly, interpretation of all these results is given to aid understanding. This interpretation has been co-produced with users and lived experience to ensure the best possible interpretation of results for a broad audience. Users are then directed to the ‘Plot’ tab to visualize the trajectories with 95% CIs (plotted alongside the mean observed scores from each occasion if desired). To further aid interpretation, we have also included the ability to calculate the outcome at a given age/time/occasion along the trajectory (i.e. depression scores at ages 13, 15, or 17), which is especially helpful for interpreting non-linear models with complex and potentially contrasting polynomial terms [15]. Finally, we also include the area under the curve (AUC), which represents the cumulative or total ‘exposure’ to the outcome over the time period being measured (i.e. how long on average individuals spend with greater levels of depression). Users can then download a summary report that includes all of this output. Statistical information supporting these methods is given in the Supplementary Materials and on the GitHub.

iii) Interactions: users can obtain group/population-specific trajectories by using the interactions tab to select between continuous and categorical variables on which to base the interactions. These interactions are based on the interaction variable of interest (i.e. sex) that is then interacted with the age/time variable to form a group-specific trajectory. Continuous variables are standardized to have a mean of zero and a standard deviation of 1, whereas categorical variables will use the level with the lowest score as the reference group. The user is then presented with all of the features stated above, but specific to interactions. Additionally, the user can make statistical comparisons between groups at given ages/times (i.e. height differences at age 12 between females and males) and between groups for the AUC (i.e. whether people with a lower socio-economic status spend longer with greater depressive symptoms). Full information is presented in the Supplementary Materials.
iv) Individual level trajectories: users can explore individual participant trajectories derived from the model that they have previously chosen (i.e. linear or quadratic polynomial models) and observe how individuals vary compared with the overall population or groups/populations from the model. Users can see how the model-predicted individual trajectories compare to the observed individual trajectories. Users can also plot ≤30 random individual trajectories from the overall population or specific to each population (i.e. 30 male trajectories and 30 female trajectories) or a specific subset of trajectories (taken from the corresponding identifier/subject variable).

## Use

We show how TIDAL can be used within the R package, although the implementation above is the same across both formats. TIDAL can be installed by using the following commands in R:

# Note: install the remotes packages if not already installed

# install.packages(“remotes”)

remotes::install_github(“TIDAL-modelling/TIDAL”)

# Note: if prompted to update packages you can select option 3/None.

library(“TIDAL”)

# Launch the R Shiny app

launchTIDAL()

# To get documentation for launchTIDAL()

? launchTIDAL

We highlight two examples of how TIDAL can be implemented by using synthetic data, which holds the same properties as real data, but without concerns over privacy [16]. The datasets used in this example can be found at https://tidal-modelling.github.io/docs/installation/synthetic_data.html. Further information about both cohorts is given in the Supplementary Materials.

### Trajectories of emotional symptoms across childhood and adolescence

The dataset in this first example comes from the Millennium Cohort Study (MCS) [17] and contains 12 720 individuals with ≤5 repeated assessments of parent-reported emotional symptoms (referred to as sdq from here) between the ages of around 3 (t1) and 14 (t5) [18]. The research question here is: how might emotional symptoms develop across childhood and through adolescence?

**Figure 3.**
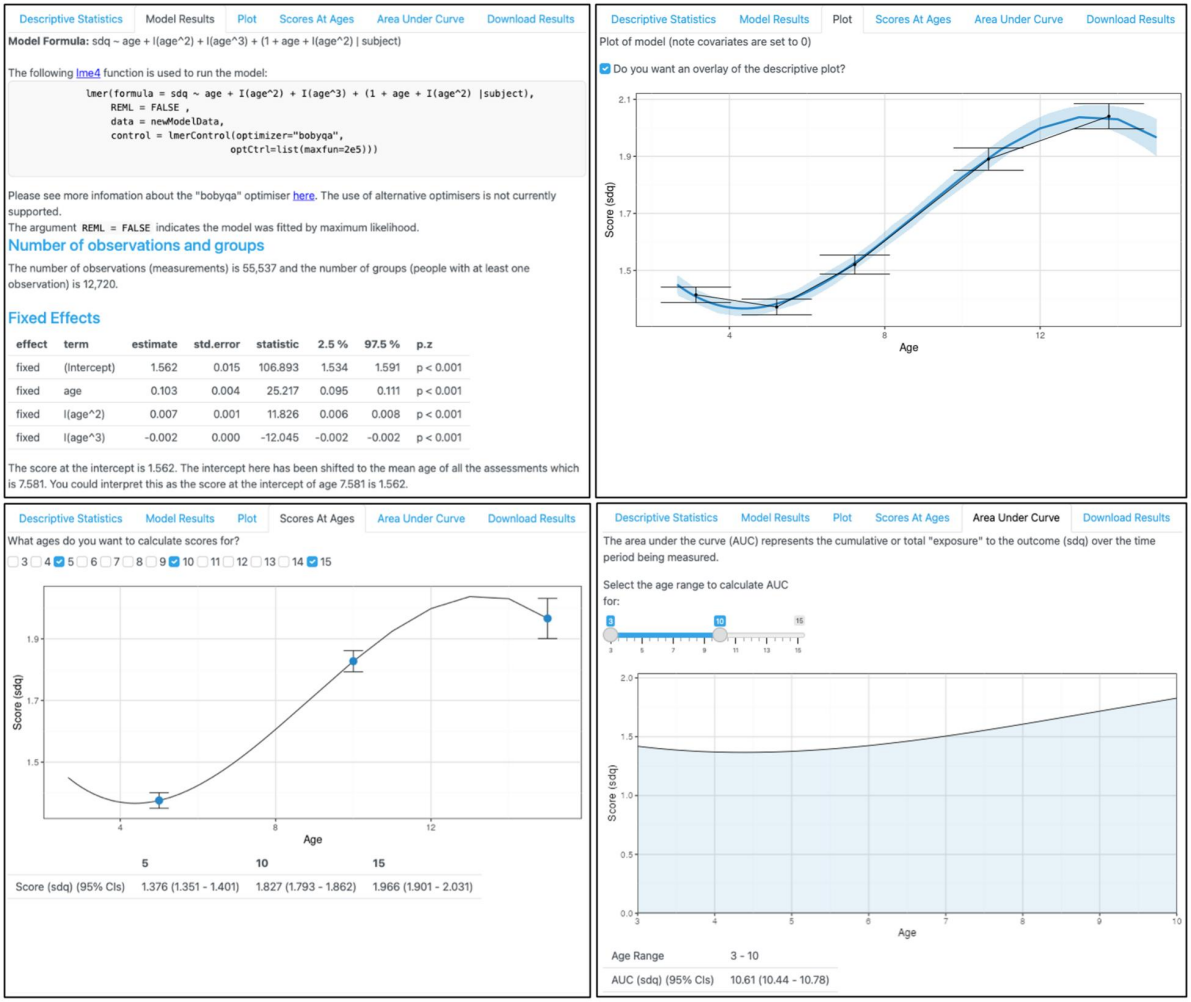
The data-exploration and analysis stage of TIDAL continued. This figure shows the output from the corresponding model from Fig. 2. Top left: the lme4 syntax used to run the model, followed by information about the number of observations and people included in the analysis, and then the table of fixed effects and interpretation of the results. Top right: the plotted sdq trajectory with 95% CIs from Fig. 2. Bottom left: the calculation of scores at different ages, with the score for the sdq calculated at ages 5, 10, and 15 shown, alongside 95% CIs. Bottom right: the area under the curve plotted and calculated for sdq trajectories between the ages of 3 and 10.

As shown in Fig. 1, the variables subject (the individual’s study ID), age_t1-age_t5 (ages at each occasion between occasions 1 and 5), and sdq_t1-sdq_t5 (sdq scores at each occasion between occasions 1 and 5) are in the original wide dataset and made into long format for analysis in TIDAL. Figure 2 then shows that the variables subject, age, occ (occasion), and sdq are carried forward from the previous stage. No confounders or covariates are included in this model. After exploring a linear model first, it appears that a cubic model may suit the data best given the non-linear pattern, so a cubic polynomial model with random intercept, linear, and quadratic terms is selected. Figure 3 shows the output from the analysis stage of TIDAL, including the lme4 syntax for the model and information about the number of observations and people. Next, the fixed and random effects are presented, showing modest effect sizes for all fixed age polynomial terms. Interpretation of these results is then given by TIDAL (not shown for brevity).

Figure 3 also shows the predicted trajectories, overlaid onto the descriptive statistics plot. The model appears to match the observed descriptive data. To further aid interpretation, TIDAL also calculates sdq scores across all ages at which age data are available (i.e. between 3 and 15 here). We can see that the predicted score at age 5 is 1.37 (95% CI: 1.35, 1.40), at age 10 is 1.82 (95%CI: 1.79, 1.86), and at age 15 is 1.96 (95% CI: 1.90, 2.03). Finally, TIDAL has calculated the AUC, which, in this example, is restricted to ages 3–10 and shows that this population has an AUC score of 10.61 (95% CI: 10.44, 10.78). Together, these analyses suggest that emotional symptoms scores tend to get higher with age, supporting similar research [15].

### Sex differences in height from childhood to young adulthood

This next example uses data from the Avon Longitudinal Study of Parents and Children (ALSPAC) [19] and contains 10 261 individuals with ≤8 repeated assessments of height between the ages of around 7 and 20. The research question here is: how do height trajectories vary between girls and boys across childhood and adolescence?

**Figure 4.**
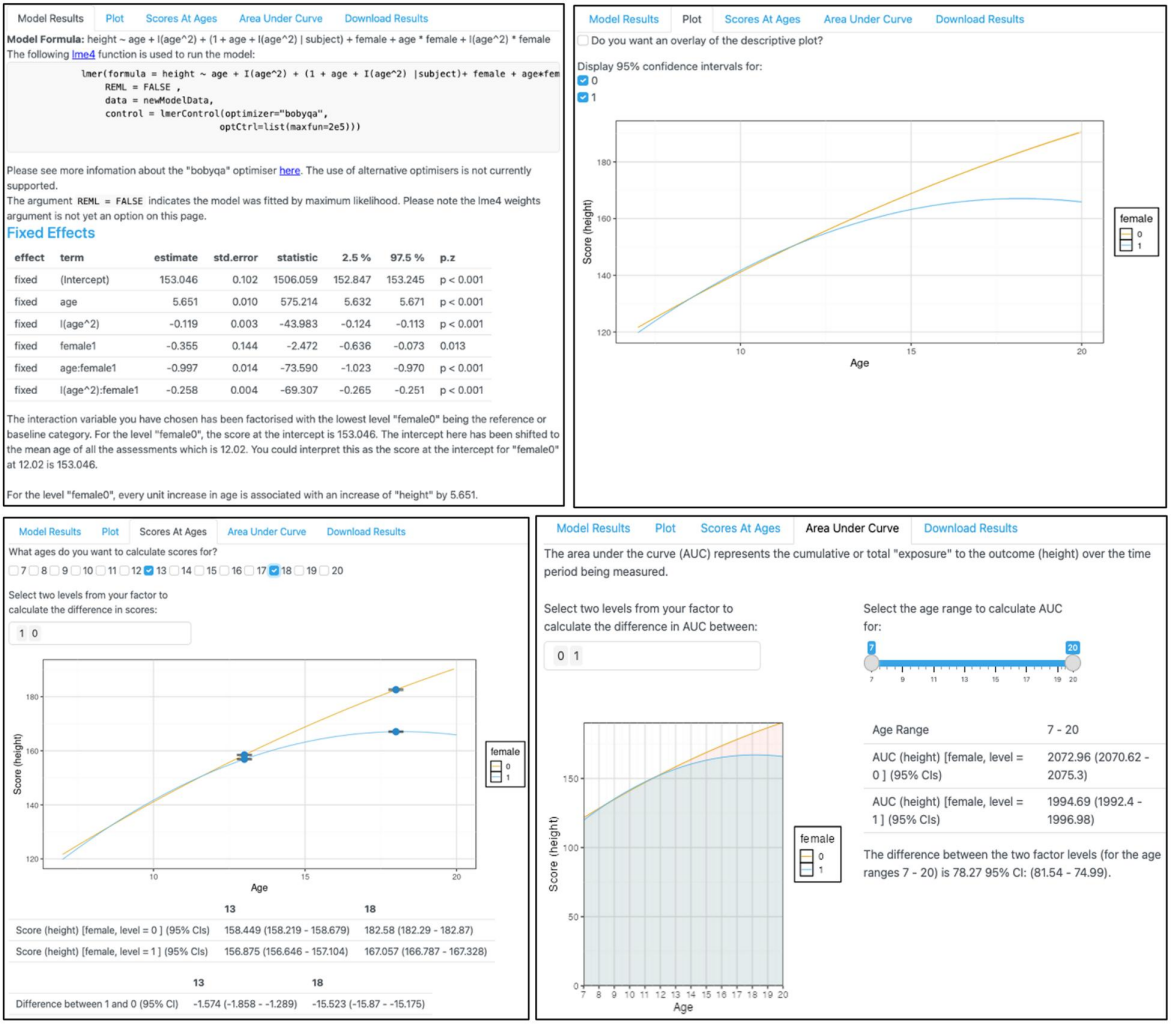
The interaction stage of TIDAL. This figure shows the output from the height-trajectories model. Top left: the lme4 syntax used to run the model, followed by information about the number of observations and people included in the analysis, and then the table of fixed effects and interpretation of the interaction results. Top right: the plotted height trajectories of girls (1) and boys (0) with 95% CIs. Bottom left: the calculation of height at different ages, with the differences in height calculated for ages 13 and 18 shown, alongside 95% CIs. Bottom right: the area under the curve (AUC) plotted and calculated for height trajectories between the girls and boys between the ages of 7 and 20, accompanied by the AUC score and 95% CIs.

For brevity, we only demonstrate analysis by using the interactions tab shown in Fig. 4, as the process up to that point will be identical to the above. Figure 4 shows large effect sizes for the fixed age terms and modest effects for the main effect of sex (variable name is female) and the interactions between sex and age terms. Interpretation of these results is then given by TIDAL (not shown for brevity).

Figure 4 shows the predicted trajectories, split by sex. Again, to further aid interpretation, TIDAL has also calculated height across all the ages at which age data are available (i.e. between 7 and 20). We can see that the predicted height at age 13 for boys is 158.44 cm (95% CI: 158.21, 158.67) and at age 18 for boys is 182.58 cm (95% CI: 182.29, 182.87), whereas, for girls, the heights at those ages are 156.87 cm (95% CI: 156.64, 157.10) and 167.05 cm (95% CI: 167.78, 167.32), respectively. TIDAL will also calculate the difference between girls and boys in those age ranges, giving a difference of –1.57 cm (95% CI: –1.85, –1.28) at age 13 and a difference of –15.52 cm (95% CI: –15.87, –15.17) at age 18. Finally, TIDAL will calculate the AUC for both girls and boys, with the difference-in-height AUC being –78.27 (95% CI: –81.54, –74.99). Together, results show that girls and boys have similar height trajectories until about the age of 12 or 13, when boys continue to grow into young adulthood and girls tend to plateau in height, as demonstrated in previous research [20].

## Discussion

TIDAL is a research tool that facilitates access, analysis, and interpretation of trajectory data. TIDAL comes in three formats, allowing users to go from raw data to visualized and interpretable trajectories in a few simple steps. A main aim of TIDAL is to remove some of the existing barriers in examining and understanding longitudinal traits and is aimed particularly at researchers, clinicians, public health experts, government analysts, and educators. Our hope is that TIDAL allows users without experience in longitudinal modelling or those without the capacity to train to make progress in understanding longitudinal traits that could develop better interventions and preventions for health conditions and wellbeing.

Longitudinal studies are becoming more essential within research. This brings challenges with how to appropriately analyse these data, especially for more complex longitudinal patterns. One of the main benefits of TIDAL is the interpretation of complex trajectories, particularly non-linear trajectories. The ability to visualize and extract information from trajectories (i.e. scores at different ages/times, AUC) is pertinent for understanding more about traits or behaviours of interest. Another benefit is the ability to build upon the provided lme4 syntax for analysis outside of TIDAL for more complex models. This includes models in which users may want to examine how a main effect (e.g. treatment) differs by other variables such as sex or social class. This requires a second-order interaction between the main effect, age/time, and sex. It is worth noting that TIDAL only uses polynomial terms to handle time/age and additional functions such as splines [5] and fractional polynomials [6] may better suit data. Future versions of TIDAL will look to include these functions of time, alongside additional features such as the age of peak velocity or the age of peak symptoms [1]. Likewise, latent class growth analysis or growth mixture modelling may be a better method to use, depending on the research question [21].

Like all research tools, TIDAL should be used responsibly and cannot mask poorly designed or inappropriate studies. We have tested TIDAL in seven longitudinal studies (including UK Biobank and Twins Early Development Study) with different outcomes and developmental stages, and found it to be robust across these datasets. However, issues may arise in studies with substantial study attrition, as in other software packages. Users should have some experience or understanding of the data that they are using, as this will help to ensure that TIDAL runs successfully and the results are robust. Additionally, users should have some foundational knowledge of mixed models to determine whether TIDAL is the appropriate tool to use and to help contextualize results. It should also be noted that TIDAL uses the lme4 package and so is limited by the power of the original R package. Alternative R packages such as ‘*nlme*’ can also be used, allowing more flexible random effects [22]. Other non-R software is also available (i.e. *MLwiN*) to fit multilevel models and accommodates a variety of estimation methods and model specifications, with the caveat that these require statistical experience and costs if outside the UK.

In conclusion, TIDAL is a user-friendly research tool designed to help users to access, analyse, and interpret trajectories data. TIDAL has all the features that users would find in published manuscripts and goes beyond much of the existing research to extract pertinent information from trajectories. We hope that TIDAL will benefit prospective users of longitudinal data and aid the interpretation and understanding of longitudinal traits.

## Ethics approval

The data collection for the MCS is approved by the UK National Health Service Research Ethics Committee. Written consent was obtained from all parents in the MCS at each survey (for MCS1, South West MREC [MREC/01/6/19]; for MCS2 and MCS3, London MREC [MREC/03/2/022, 05/MRE02/46]; for MCS4, Yorkshire MREC [07/MRE03/32]; for MCS5, Yorkshire and The Humber-Leeds East [11/YH/0203]; for MCS6, London MREC [13/LO/1786]; for MCS7, North East–York [REC ref 17/NE/0341]). Ethical approval for the study was obtained from the ALSPAC Ethics and Law Committee and the Local Research Ethics Committees.

## Supplementary Material

dyaf095_Supplementary_Data

## Data Availability

Further information and data access to the Millennium Cohort Study can be found at https://cls.ucl.ac.uk/cls-studies/millennium-cohort-study/. Further information and data access to the Avon Longitudinal Study of Parents and Children study can be found at http://www.bristol.ac.uk/alspac/researchers/our-data/.
